# Rectopexy for Rectal Prolapse

**DOI:** 10.3389/fsurg.2015.00054

**Published:** 2015-10-19

**Authors:** Nasra N. Alam, Sunil K. Narang, Ferdinand Köckerling, Ian R. Daniels, Neil J. Smart

**Affiliations:** ^1^Exeter Surgical Health Services Research Unit (HeSRU), Royal Devon and Exeter Hospital, Exeter, UK; ^2^Department of Surgery, Center for Minimally Invasive Surgery, Academic Teaching Hospital of Charité Medical School, Vivantes Hospital, Berlin, Germany

**Keywords:** ventral mesh rectopexy, mesh rectopexy, pelvic organ prolapse, biological mesh, vMR

## Abstract

**Introduction:**

Ventral mesh rectopexy (VMR) is a recognized treatment for posterior compartment pelvic organ prolapse (POP). The aim of this review is to provide a synopsis of the evidence for biological mesh use in VMR, the most widely recognized surgical technique for posterior compartment POP.

**Methods:**

A systematic search of PubMed was conducted using the search terms “VMR,” “ventral mesh rectopexy,” or “mesh rectopexy.” Six studies were identified.

**Results:**

About 268/324 patients underwent ventral rectopexy using biological mesh with a further 6 patients having a combination of synthetic and biological mesh. Recurrence was reported in 20 patients; however, 6 were from studies where data on biological mesh could not be extracted. There are no RCTs in VMR surgery and no studies have directly compared types of biological mesh. Cross-linked porcine dermal collagen is the most commonly used mesh and has not been associated with mesh erosion, infection, or fistulation in this review. The level of evidence available on the use of biological mesh in VMR is of low quality (level 4).

**Conclusion:**

Ventral mesh rectopexy has become prevalent for posterior compartment POP. The evidence base for its implementation is not strong and the quality of evidence to inform choice of mesh is poor.

## Introduction

**Figure 1:**
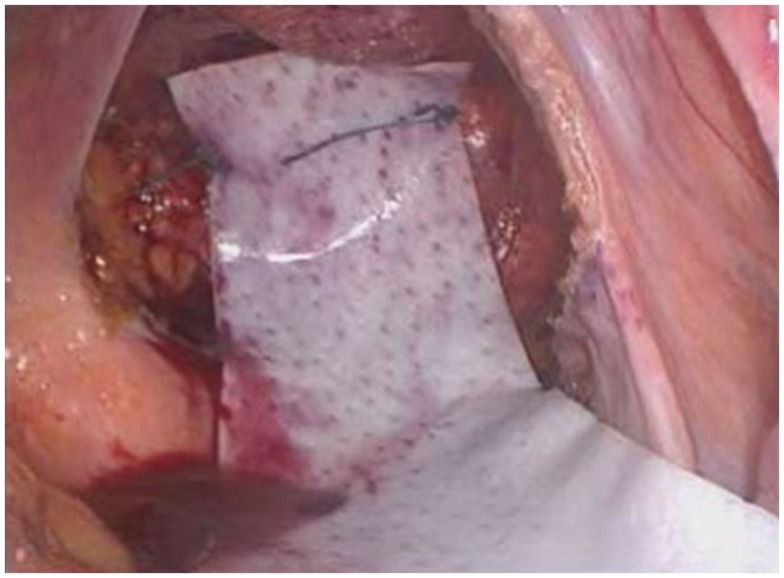
**Placement of mesh anterior to rectum and suturing to the anterior wall of the rectum ± suture to vaginal vault**.

**Figure 2:**
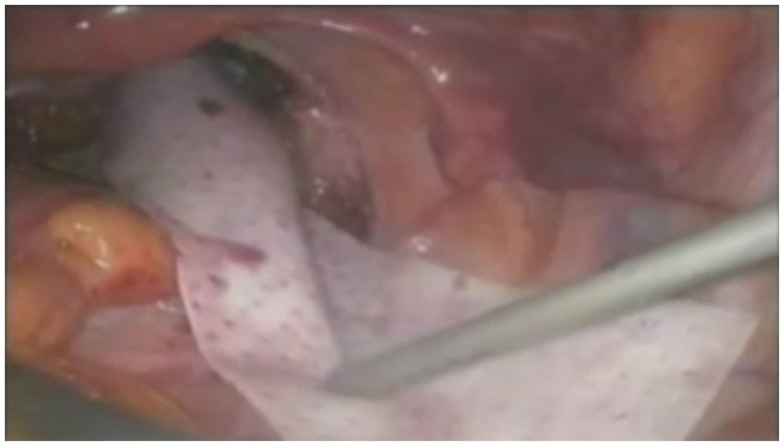
**Tacking of mesh to sacral promontory**. Photographs by kind permission of Mr. Mark Mercer-Jones, Consultant Colorectal Surgeon, Gateshead, UK.

Ventral mesh rectopexy (VMR) is a recognized treatment for posterior compartment pelvic organ prolapse (POP). It is believed to address functional bowel symptoms by providing suspensory support to the prolapsing organ (in this case the rectum ± the vaginal vault) and avoiding the autonomic denervation that results in *de novo* symptomatology. Consequently, it improves obstructive defaecatory symptoms as well as symptoms of incontinence (1–4) without initiating significant new onset constipation (1, 5). VMR comprises dissection of the rectovaginal septum from above to the level of the pelvic floor. This is followed by fixation of a synthetic or biological prosthesis to the anterior wall of the rectum and proximally to the sacral promontory (Figures 1 and 2). The vaginal vault may also be fixed to the mesh to provide support and help obliterate the deep rectovaginal pouch. VMR has rapidly established itself in Europe as the procedure of choice for posterior compartment POP in spite of a limited evidence base.

A variety of synthetic meshes have been used for a wide range of POP surgery but there have been reports of high rates of pelvis sepsis, as well as concerns regarding mesh erosion, dyspareunia, fistulation. and stricturing (6–8). The Food and Drug Administration (FDA) issued a warning in 2011 that, *“serious complications associated with surgical mesh for transvaginal repair of POP are not rare”* (9). It is not clear to what extent this warning is relevant to POP surgery carried out via abdominal approaches. Nevertheless, since it has been postulated that biological mesh may cause fewer complications in comparison to synthetic mesh in certain high-risk circumstances (10–12). This has led to an increase in the popularity of biological mesh use for POP surgery. The aim of this review is to provide a synopsis of the evidence for biological mesh use in VMR, the most widely recognized surgical technique for posterior compartment POP.

## Methods

A systematic search of PubMed was conducted using the search terms “VMR,” “ventral mesh rectopexy,” or “mesh rectopexy.” Titles, abstracts, and finally full texts were analyzed for studies reporting on the use of biological mesh in rectopexy. Inclusion criteria were studies that described a ventral rectopexy using a biological mesh in either an open or laparoscopic technique. Studies were excluded if only synthetic mesh was used or if there was no mention of a mesh. Furthermore, studies on patients under the age of 18 were excluded as well as non-English language studies, technical tips, or duplicates series from the same research group. Overall, the search yielded six studies for analysis after the exclusion of review articles. The study characteristics are presented (Table 1).

**Table 1:** **Study characteristics**.

Author (year)	Study design	No. of pts	Age	Sex (M:F)	Patient characteristics	Material used	Intervention	Follow-up (months)	Recurrence	Complications	LoE	Notes
Enríquez-Navascués et al. (23)	Case Series	57	Mean: 66 (19–81)	2:55	Total rectal prolapse: 11	Acellular porcine dermis biological mesh (Pelvicol^®^): 4 polypropylene macroporous synthetic mesh (Ginemesh^®^, Ethicon): 4 Combination: 3	Laparoscopic rectopexy	25 (4–48) Median	1 (Biologic)	1 reoperation	4	
					Rectoenteroceles with or without descending perineal syndrome: 4	Pelvicol^®^: 1 Combination: 3	Laparoscopic rectopexy			-	
					Genitourinary pelvic organ prolapse: 42	Pelvicol^®^: 36 Ginemesh^®^: 6	Pfannenstiel: 31 Laparoscopic: 11		9 (Biologic)	4 reoperation	

Wahed et al. (24)	Case series	65	62 (31–89) Median	3:62	Full thickness rectal prolapse: 27 rectocele with obstructive defecation symptoms: 23 vaginal vault prolapse: 14 Fecal Incontinence: 1	Permacol^™^	Lap ventral rectopexy	12 (1–29) Median	2	Diarrhea: 2	4	
										UTI: 1	
										MI: 1	
										Sacral osteomyelitis: 1	
										Intersphincteric abscess: 1	
										Port site pain: 2	
										Strangulated port site hernia: 1	

Sileri et al. (25)	Case Series	34	59 (5–78) median	0:34	Grade III or IV rectal prolapse	Permacol^™^	Lap ventral rectopexy	12 months (6–28) mean	2	SBO: 1	4	
										UTI: 4	
										Subcutaneous emphysema: 2	
										Sacral pain: 1	
										Hematoma: 1	

Powar et al. (26)	Case series	120	62.5 years (25–93)	0:120	Rectocele and internal prolapse: 57 Full-thickness rectal prolapse: 53	Surgisis Biodesign^©^ : 89 Non-absorbable polypropylene mesh: 31	Lap ventral rectopexy	7.6 months median	3 (Bio mesh)	Biologic group: exacerbation of chronic pain: 3	4	Cannot separate out pts who had Surgisis^©^
										Lumbar discitis: 1	
					Other (solitary rectal ulcer): 3					Pelvic pain: 2	
										Post-operative hypotension: 1	
										Port site pain: 1	
										Vaginal discharge: 1	
										Nausea: 1	
										Urinary retention: 1	
										Atelectasis: 1	

Evans et al. (27)	Case Series	36 (30 surgery)	44 (15–81) median	5:31	SRUS: obstructive defecation: 36 Clinical external rectal prolapse: 4 External prolapse: 10 Internal rectal prolapse Grade I: 2(6%), Grade III: 6 (17%), Grade IV: 14 (39%)	Polypropylene: 27 Permacol^™^: 3	Laparoscopic ventral mesh rectopexy: 29 STARR: 1	36 months (3–78) Median	3 (unknown whether related to Biological mesh)	Vaginal stitch sinus: 1 Wound infection: 1 Port site hernia: 1 Mortality: 1	4	Cannot separate out 3 pts who had Permacol^™^

Sileri et al. (28)	Case series	12	Mean age 63 years, range 23–78)	0:12		Permacol^™^	Lap ventral rectopexy	5 months	Not reported	Port site hematoma: 1 Subcutaneous emphysema: 1	4	

## Results

In the 6 case series, there was a total of 324 patients. Of these, 268 patients underwent ventral rectopexy using biological mesh with a further 6 patients having a combination of synthetic mesh and biological mesh. Overall, 155 patients underwent VMR using additionally cross-linked porcine dermal collagen (Permacol™ or Pelvicol™) and 89 using porcine intestinal submucosa (Surgisis^©^). Recurrence was reported in 20 patients; however, 6 of these were from studies where data on biological mesh could not be extracted. One study did not report recurrence. Complications are outlined (Table 1).

There are no randomized controlled trials in VMR surgery generally and no studies have directly compared types of biological mesh, e.g., cross-linked vs. non-cross-linked. Cross-linked porcine dermal collagen is the most commonly used mesh and has not been associated with mesh erosion, infection, or fistulation in this current review. The level of evidence available on the use of biological mesh in VMR is of low quality (level 4) (13).

## Discussion/Summary

Ventral mesh rectopexy has become established as the current procedure of choice for posterior compartment POP without a high quality evidence base in support of its adoption and therefore this has consequently been called into question (14). In light of the limited evidence base for VMR generally, it is perhaps of no surprise that the level of evidence for any specific mesh type, either synthetic or biological, is level 4. The expert consensus assumes that VMR is the optimal treatment paradigm in many circumstances (15). This may well turn out to be the case, but as yet the evidence basis is lacking and recommendations regarding any specific type of mesh are at best grade C (16).

All the included studies are retrospective, often with short follow-up, have small numbers of patients and are usually derived from single institutions. The applicability of the findings to a wider population is uncertain. There is one comparative case series with 29 patients undergoing laparoscopic VMR using a biological mesh and 29 patients matched for age and surgical indication, undergoing laparoscopic VMR using a synthetic mesh (17). However, it did not meet the inclusion criteria for the review as it was a subset analysis of data that has already been presented and discussed and was therefore excluded. Furthermore, the other key limitation for most of the included studies is the variability of outcome reporting and the lack of standardization of outcome measures. Some studies report functional outcome scores for both constipation and incontinence, e.g., Wexner/FISI, but these scoring systems are not necessarily appropriate for obstructed defaecation syndrome (ODS) or prolapse (18, 19). Disease-specific scoring systems such as pelvic organ prolapse quantification system (POP-Q) or the ODS score (20), and quality of life scores (e.g., SF-36 EQ-5D) may be more appropriate but, these have not been used in any of the studies included in this review. Anorectal physiology results are reported in some studies but correlation to anatomy, recurrence or symptomology is not clearly defined. For those studies where VMR was used to treat ODS, post-operative defaecography that supports long-term anatomical correction of prolapse has not been reported.

Complications in the included studies are inconsistently reported and standardized methods of reporting, such as Clavien–Dindo have not been used (21). Two studies did not meet the inclusion criteria because they only addressed complications pertaining to VMR. The first was a systematic review of reported complications, which failed to demonstrate any difference in complications between synthetic and biological mesh although the follow-up was short (22). The second study has reported 50 patients referred for complications following VMR and has documented operative strategies and techniques. Although complications from both biological and synthetic meshes are discussed, there is no denominator provided and therefore it is not possible to ascertain the relative frequency of complications with each type of mesh (6). It is interesting to note that the concerns raised by the FDA have not been reported in the literature pertaining to VMR to the same extent. Although most series have follow-ups of short duration, in the transvaginal approach mesh complications were mainly reported within 12 months (8). This suggests that the concerns relating to mesh placement via the transvaginal or other perineal approaches may not be extrapolated to transabdominal approaches.

## Conclusion

Ventral mesh rectopexy has become prevalent for posterior compartment POP. The evidence base for its implementation is not strong and the quality of evidence to inform choice of mesh is poor.

## Conflict of Interest Statement

The authors declare that the research was conducted in the absence of any commercial or financial relationships that could be construed as a potential conflict of interest.
